# A novel deep learning method to segment parathyroid glands on intraoperative videos of thyroid surgery

**DOI:** 10.3389/fsurg.2024.1370017

**Published:** 2024-04-19

**Authors:** Tian Sang, Fan Yu, Junjuan Zhao, Bo Wu, Xuehai Ding, Chentian Shen

**Affiliations:** ^1^ School of Computer Engineering and Science, Shanghai University, Shanghai, China; ^2^ Department of Nuclear Medicine, Shanghai Sixth People’s Hospital Affiliated to Shanghai Jiao Tong University School of Medicine, Shanghai, China; ^3^ Department of Thyroid, Breast and Hernia Surgery, Shanghai Sixth People’s Hospital Affiliated to Shanghai Jiao Tong University School of Medicine, Shanghai, China

**Keywords:** artificial intelligence, deep learning, parathyroid glands, intraoperative videos, localization, segmentation

## Abstract

**Introduction:**

The utilization of artificial intelligence (AI) augments intraoperative safety and surgical training. The recognition of parathyroid glands (PGs) is difficult for inexperienced surgeons. The aim of this study was to find out whether deep learning could be used to auxiliary identification of PGs on intraoperative videos in patients undergoing thyroid surgery.

**Methods:**

In this retrospective study, 50 patients undergoing thyroid surgery between 2021 and 2023 were randomly assigned (7:3 ratio) to a training cohort (*n* = 35) and a validation cohort (*n* = 15). The combined datasets included 98 videos with 9,944 annotated frames. An independent test cohort included 15 videos (1,500 frames) from an additional 15 patients. We developed a deep-learning model Video-Trans-U-HRNet to segment parathyroid glands in surgical videos, comparing it with three advanced medical AI methods on the internal validation cohort. Additionally, we assessed its performance against four surgeons (2 senior surgeons and 2 junior surgeons) on the independent test cohort, calculating precision and recall metrics for the model.

**Results:**

Our model demonstrated superior performance compared to other AI models on the internal validation cohort. The DICE and accuracy achieved by our model were 0.760 and 74.7% respectively, surpassing Video-TransUnet (0.710, 70.1%), Video-SwinUnet (0.754, 73.6%), and TransUnet (0.705, 69.4%). For the external test, our method got 89.5% precision 77.3% recall and 70.8% accuracy. In the statistical analysis, our model demonstrated results comparable to those of senior surgeons (senior surgeon 1: *χ*^2^ = 0.989, *p* = 0.320; senior surgeon 2: *χ*^2^ = 1.373, *p* = 0.241) and outperformed 2 junior surgeons (junior surgeon 1: *χ*^2^ = 3.889, *p* = 0.048; junior surgeon 2: *χ*^2^ = 4.763, *p* = 0.029).

**Discussion:**

We introduce an innovative intraoperative video method for identifying PGs, highlighting the potential advancements of AI in the surgical domain. The segmentation method employed for parathyroid glands in intraoperative videos offer surgeons supplementary guidance in locating real PGs. The method developed may have utility in facilitating training and decreasing the learning curve associated with the use of this technology.

## 1 Introduction

The parathyroid gland (PG) is the smallest endocrine organ in the human body and plays an important role in maintaining the balance of calcium metabolism. Identifying and safeguarding PGs constitute a pivotal aspect of thyroid surgery. Given their small size and close anatomical proximity to lymph nodes or adipose tissues, there exists a risk of PG damage and compromised blood supply during thyroid surgery. Unfamiliarity with the morphology and anatomy of PGs heightens the susceptibility to damage, resulting in hypocalcemia that adversely affects the patient's quality of life. The injury of PGs can easily lead to postoperative hypocalcemia and other complications. One of the most common complications after total thyroidectomy is hypoparathyroidism. According to existing research, the incidence rate is about 30%–60% (1–3). It has been reported that the incidence of accidental parathyroid removal is up to 12%–28% (4). The symptoms of PG injury mainly include hand and foot twitching, limb sensory abnormalities, muscle spasms, and even life-threatening conditions.

Distinguishing PGs from similar tissues like thyroid, lymph nodes, or brown adipose tissues poses a significant challenge. Although all surgeons learn to differentiate PGs from other tissues, the length of this learning curve may be different for each surgeon, depending on experience. Even experienced surgeons cannot guarantee that all PGs will be recognized and protected in every thyroid surgery. At present, there are some methods to recognize PGs, such as carbon nanoparticles negative development, parathyroid hormone (PTH) in fine-needle aspiration (FNA) washout fluids, pathology validation, or near-infrared autofluorescence (NIRAF) (5–7). However, these methods have some limitations, such as longer periods of detection, high costs, false-positive results. Therefore, the existing recognition skills of PGs still rely on experienced surgeons. Nevertheless, early recognition of PGs before dissection is very helpful to guide dissection. In this case, a visual algorithm that recognizes the shapes and localization of a PG in the surgical field based on deep learning would be useful to shorten the learning curve and guide dissection.

To address those issues, we proposed the Video-Trans-U-HRNet model. At present, video AI technology has developed rapidly and is widely used in the medical field, such as robot surgery (8). Our task is to locate and segment the PG during thyroidectomy. To our knowledge, the application of AI methods to the localization and segmentation of PGs has mainly been limited to static images. However, thyroid surgery is a dynamic process that requires the full attention of the surgeon, and intraoperative videos can better reflect the real situation of the PG during surgery. Furthermore, the position and shape of PGs can undergo deformation and displacement due to the surgeon's manipulation, emphasizing the need for automatic localization and segmentation during the procedure. This not only enhances precision but also helps prevent accidental injury to PGs by the surgeon. In thyroid surgery, the PGs exhibit similarities to many other tissues, creating a challenge for existing AI models employed in medical segmentation and detection tasks to accurately recognize them. The objective of our study is to test a new AI-based method for recognizing PG and compare its performance to both junior and senior surgeons, with the ultimate aim of enhancing patient safety.

## 2 Methodology

### 2.1 Developing the AI model

**Figure 1:**
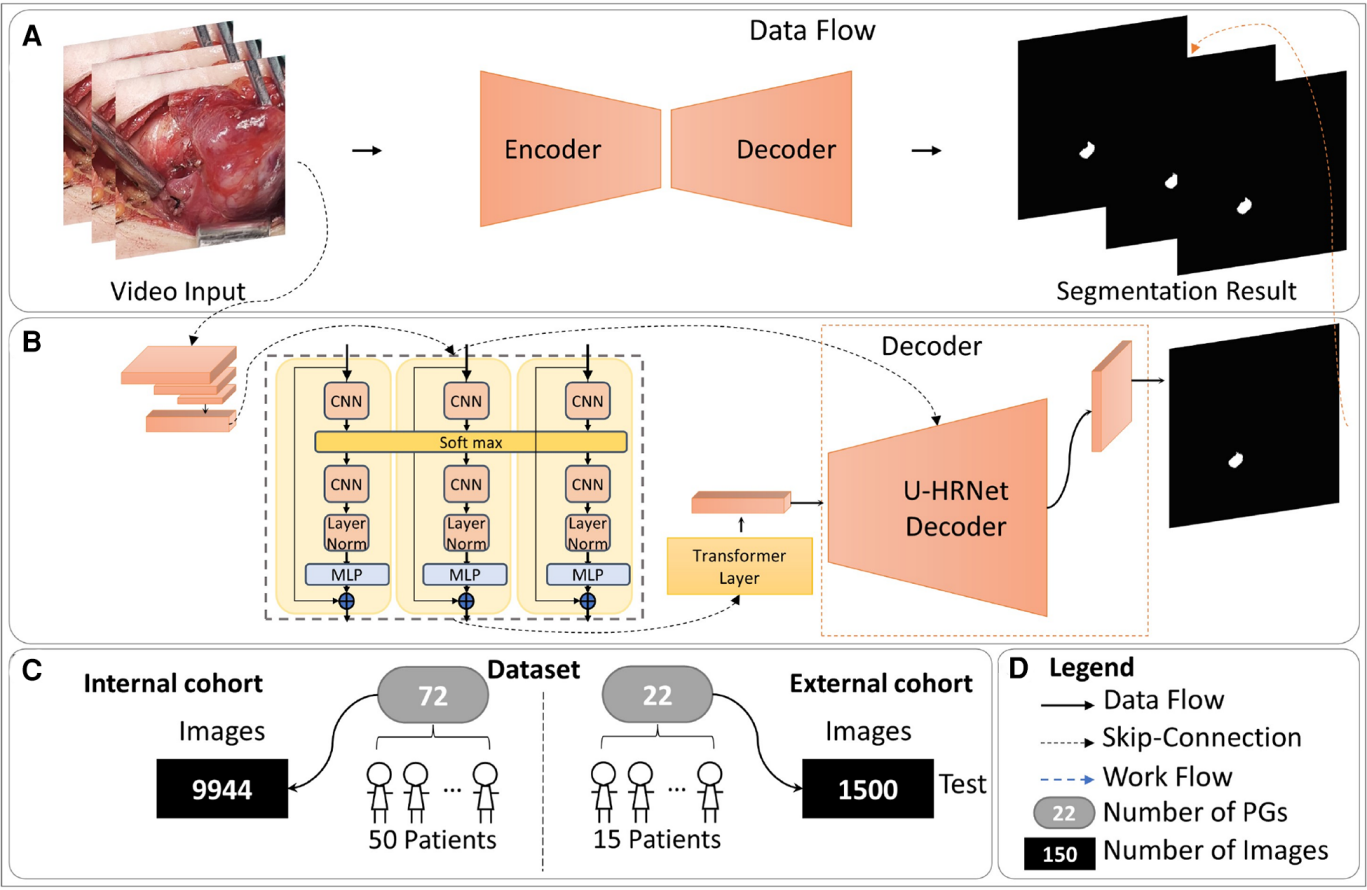
The overall of proposed method. (**A**) The overall data flow of our method. (**B**) The detailed structure of our AI model, consisting of the Temporal Contextual Module (TCM), Encoder part, and Decoder part. (**C**) The dataset employed in this study. (**D**) The legends used in this figure.

The entire method is illustrated in Figure 1, comprising four parts: (A) The overall data flow of our method. (B) The detailed structure of our AI model, consisting of the Temporal Contextual Module (TCM), Encoder part, and Decoder part. (C) The dataset employed in this study. (D) The legends used in this figure. Zeng et al.'s work demonstrated the applicability of classical semantic segmentation models in medical segmentation. Drawing inspiration from their Video-TransUNet, we devised a deep-learning segmentation method tailored for the specific task of PGs video segmentation (9). The TransUnet model demonstrates robust performance in organ segmentation tasks, achieving outstanding results across diverse public datasets (10). Building upon TransUnet, we introduced a TCM to establish links between contextual information across frames for video segmentation (11). As depicted in Figure 1B, the TCM module comprises three parallel frames processing modules, all sharing a softmax layer. This module adeptly captures information from both preceding and succeeding frames, skillfully integrating it for feature extraction of the current key frame.

**Figure 2:**
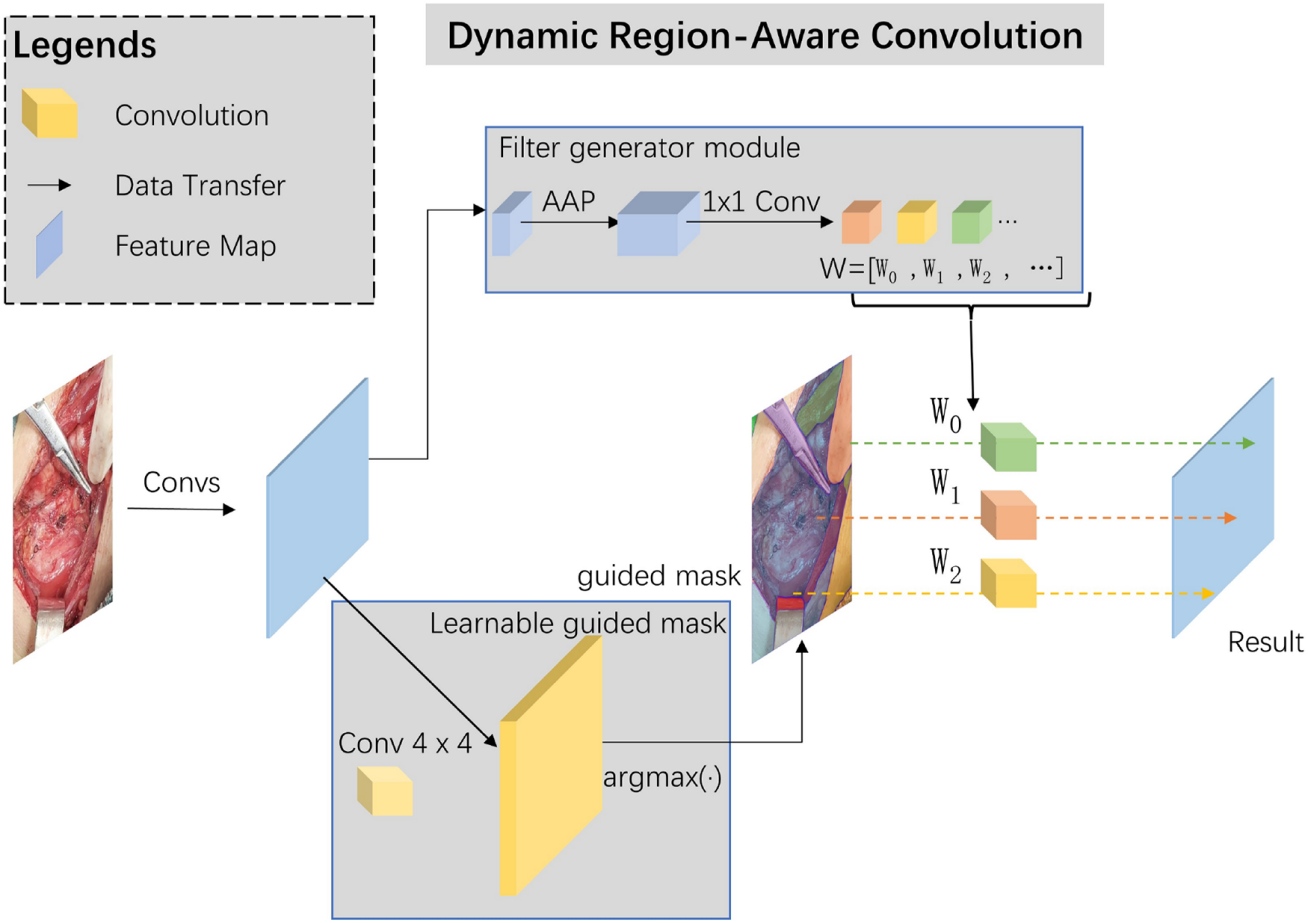
The specific structure of dynamic region-aware convolution.

Skip-connections in U-shape net are crucial in multi-scale features transfer and fusion, we added a dynamic region-aware convolution (DRConv) module into skip-connection for preliminary extraction of multi-scale features (12). The specific module structure and our implementation details are shown in Figure 2.

**Figure 3:**
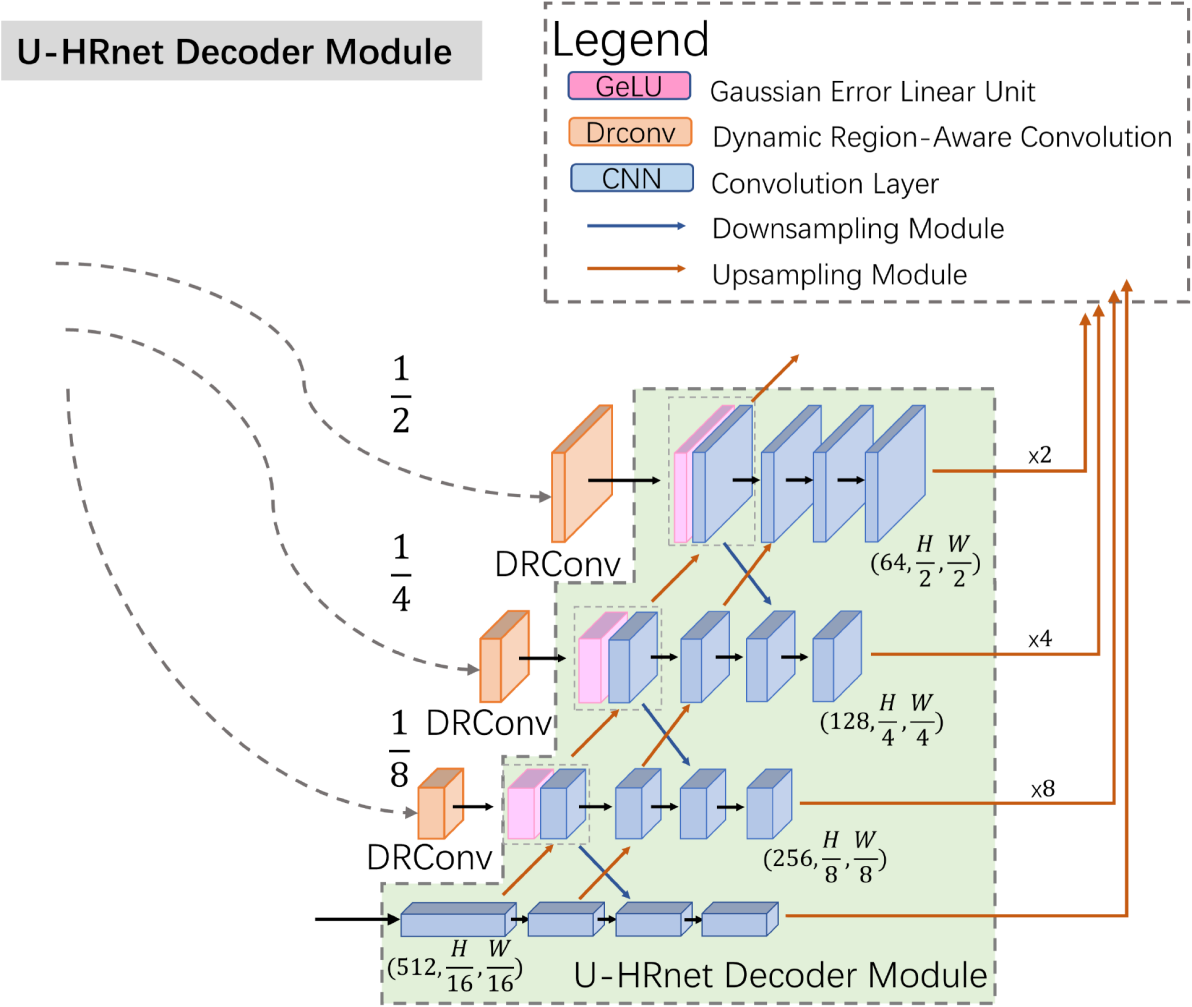
The specific structure of U-HRNet decoder module.

To improve the communication of high-level semantic information and enhance the integration of low-level features, we modified the decoder structure based on Wang's work, as illustrated in Figure 3 (13). In contrast to the TransUnet decoder, the U-HRNet Decoder is more intricate, featuring additional feature transfer paths between adjacent layers and more fusion components. This redesign is geared towards achieving more accurate segmentation results. To align with reality, we implemented a post-processing method that excludes prediction results smaller than 50 × 50 pixels in size.

### 2.2 Patients and surgical technique

A prospective analysis was conducted on a total of 65 patients undergoing thyroid surgery at Shanghai Sixth People's Hospital from August 24, 2021, to June 17, 2023. Approval for this study was obtained from the Institutional Ethics Review Board and the Ethics Committee of Shanghai Sixth People's Hospital [Approval no. 2022-KY-178 (K)].

During surgery, a high-resolution camera was used to take videos of the wound surface, with the lens placed 15 cm away from the surgical field. Each patient underwent the recording of 1–2 videos. Each PG in videos was validated by the immune colloidal gold technique (ICGT) (5). A total of 116 videos, comprising 11,444 frames, were collected. Each video spanned 5–10 s, containing 50–100 frames. Within the internal cohort, 101 videos with 9,944 frames were meticulously labeled based on pathological results. In contrast, the external cohort consisted of 15 videos with 1,500 frames, serving solely for evaluation purposes and lacking specific segmentation labeling. Specific patient characteristics were shown in Table 1.

**Table 1:** Patient characteristics.

Parameter	Value
Age, year, median (IQR)	47 (21.5)
Sex, *n* (%)	
Female	47 (72.3)
Male	18 (27.7)
Diagnosis, *n* (%)	
Thyroid cancer	48 (73.8)
Thyroid nodule	12 (18.5)
Graves’ disease	5 (7.7)
Procedure, *n* (%)	
Total thyroidectomy	28 (43.1)
Thyroid lobectomy	37 (42.1)
Hospital duration, day, median (IQR)	5 (3.5)
Operation time, min, median (IQR)	70 (35)
Parathyroid Glands	
Training	52
Internal Validation Cohort	20
Independent Test Cohort	22

Parathyroid glands represent the number of parathyroids used for model training.

### 2.3 Dataset and comparison method

During this process, a senior surgeon with over 20 years of experience labeled all the PGs in videos using labelme software, including the location and contour of the PGs. The video data from 50 patients were allocated for internal training and validation sets, whereas the remaining data from 15 patients constituted the independent external validation cohort. The intersection over union (IoU) threshold selected for comparing prediction results with ground truth was set at 0.5. Performance evaluation of the deep learning model was based on the overlap in prediction masks generated by the model and those manually placed by the research team on each frame. Precision and recall were calculated for the study. Precision of deep learning models is similar to “positive predictive value,” and attempts to answer what proportion of positive classifications was actually correct. The specific calculations were placed in Section 2.4. On the other hand, recall measures the model's ability to detect positive samples, similar to “sensitivity,” and was calculated as the ratio between the number of positive samples correctly classified as positive to the total number of positive samples. Two levels of surgeons evaluated the videos in the external validation cohort. Two levels of surgeons include 2 surgeons with more than 10 years of clinical experience in thyroidectomy and 2 surgeons with less than 10 years of clinical experience. They represented senior surgeons and junior surgeons respectively. All surgeons participating in the study had received specialist training in thyroid surgery. Our participating surgeons—both junior and senior—perform an average of 600 thyroid and parathyroid surgeries annually. This also includes the necessary training and assessments relevant to these procedures. Surgeons figured out the possible location of PGs in the video. Precision, recall, and accuracy were calculated. We compared the performance of the AI model and 2 levels of surgeons.

### 2.4 Evaluation metrics

For the evaluation of the AI model, we employed AI evaluation metrics. In this section, we introduced an evaluation metric specifically designed for PGs identification. Positive samples were calculated based on samples with an IoU threshold greater than 50% with the Ground Truth. Given that parathyroid glands are instances, segmentation results with an IoU of more than 50% were considered reasonable in the medical field. During the inference process, our method was evaluated in both the internal validation set and the external test cohort. Precision, recall, and accuracy were abbreviated as PRE, REC, and ACC, respectively. True positive, false positive, false negative, and true negative were abbreviated as TP, FP, FN, and TN, respectively. Specific computations are shown below.

Precision=TP/(TP+FP)

Recall=TP/(TP+FN)

Accuracy=(TP+TN)/(TP+FN+FP+TN)

Dice coefficient and Jaccard coefficient is the most commonly used evaluation metric in medical segmentation field, and the formulas are as follows. While *X* represents the area of the Ground Truth, *Y* represents the area of Prediction results. Both Dice and Jaccard may evaluate how well the prediction results cover the Ground Truth. Jaccard is abbreviated as JAC.

Dice=2×(|X∩Y|)/(|X|+|Y|)

Jaccard=(|X∩Y|)/(|X|+|Y|−|X∩Y|)

For evaluation of video, we proposed a new coefficient for frame loss performance. Loss Frames represented the number of frames with no target. Total Frames represented the total number of frames. The formula is as follows.

FLR=(LossFrames)/(TotalFrames)

A *χ*^2^ test was used to compare the PG recognition rate between the AI model and different groups of surgeons. Comparisons between groups were statistically processed by SPSS 26.0, and statistical significance was assigned for *p* values < 0.05.

## 3 Results

### 3.1 Internal validation results

We conducted a comparative analysis of our method against three advanced deep-learning methods applied in the relevant medical field using our validation dataset. The results are presented in Table 2. As shown in Table 2, our proposed method outperformed the others in the validation dataset, indicating its superior performance. In internal validation cohort, we got 84.9% Precision and 81.3% Recall.

**Table 2:** Result of proposed method.

Model	DICE	ACC	PRE	REC	hd_95	JAC	FLR
Our method	0.760	74.7%	80.2%	82.4%	28.44	0.769	11%
Video-TransUnet	0.710	70.1%	76.2%	71.4%	18.49	0.677	15%
Video-SwinUnet	0.754	73.6%	84.8%	80.4%	19.22	0.714	12%
TransUnet	0.705	69.4%	76.2%	74.3%	19.98	0.650	20%

### 3.2 Comparison with surgeons

**Figure 4:**
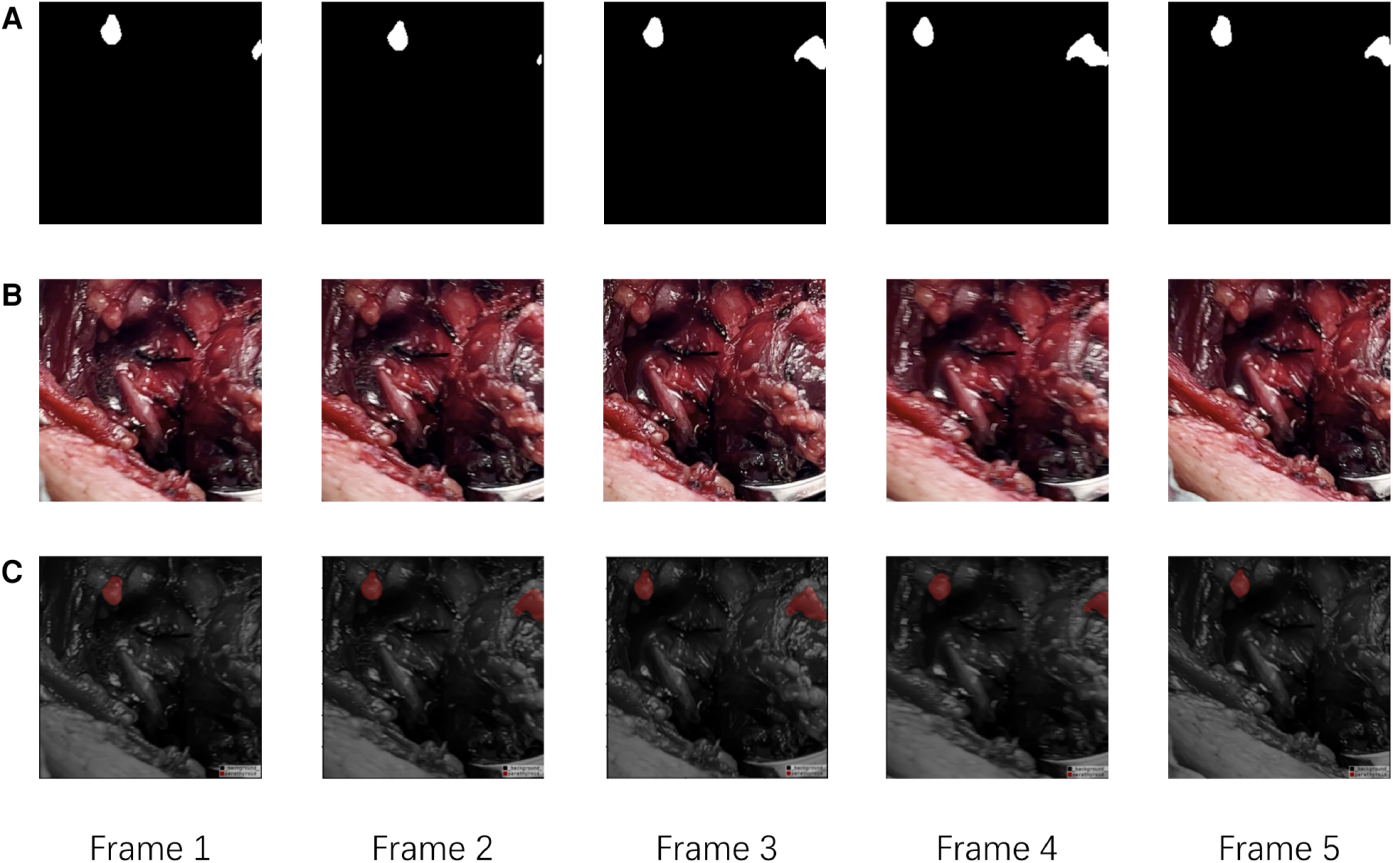
The visualization of evaluation results of our method. (**A**) The Ground Truth of PGs in continues frames. (**B**) The raw continues frames. (**C**) The predict results of our model.

For external validation, our method was compared with two different levels of surgeons, junior surgeons and senior surgeons. As the external cohort lacked specific labels, frames in the external validation dataset only had coarse labels, making accuracy evaluation metrics impractical for this cohort. Detailed comparison results are presented in Table 3. In this comparison, our method achieved a precision of 89.5% and an accuracy of 70.8% in identifying PGs, outperforming all other surgeons. In comparison to all surgeons, our method achieved higher precision results as it tends to provide more accurate outcomes, while surgeons leaned towards offering more candidates to avoid missing PGs. Despite surgeons having more prior information, such as potential PG locations and their numbers, both the accuracy and precision of our method surpassed those of the surgeons. Comparison with senior surgeons revealed that surgeons with extensive experience identified more accurate PGs.The results suggest that the AI model can assist in providing more accurate PG results, thereby reducing the time and effort required for surgeons to identify PGs. Additionally, our method, based on a video recognition algorithm, demonstrates the ability to track PGs throughout the entire process of thyroidectomy, as depicted in Figure 4.

**Table 3:** Result of proposed method.

Model	PRE	REC	ACC
Our method	89.5%	77.3%	70.8%
Junior surgeon 1	75.0%	81.8%	64.3%
Junior surgeon 2	80.0%	72.7%	64.0%
Senior surgeon 1	70.0%	63.6%	50.0%
Senior surgeon 2	84.6%	84.6%	45.8%

In addition, we compared the differences between our model and two levels of surgeons in the external cohort. Our AI model demonstrated results comparable to those of senior surgeons, with no significant difference (senior surgeon 1: *χ*^2^ = 0.989, *p* = 0.320; senior surgeon 2: *χ*^2^ = 1.373, *p* = 0.241). And our model was superior to two junior surgeons, with a statistically significant difference (junior surgeon 1: *χ*^2^ = 3.889, *p* = 0.048; junior surgeon 2: *χ*^2^ = 4.763, *p* = 0.029).

## 4 Discussion

In our retrospective study, we evidenced the proficiency of the proposed AI model in the recognition of PG in intraoperative videos. This model achieved a performance level demonstrating equivalence to that of two senior surgeons. A comparative analysis with multiple surgeons further solidified the potential of our presented method, indicating its application could bolster intraoperative recognition of PG. Ultimately, the implementation of this methodology could precipitate a meaningful advancement in surgical accuracy, thereby substantially augmenting patient safety measures. To the best of our knowledge, this marks the inaugural instance of an AI model predicting the location and masks of PGs through the analysis of intraoperative videos in patients undergoing thyroidectomy.

AI in medicine holds the potential for expedited and standardized training. Kitaguchi et al. demonstrated the application of convolutional neural networks for automatic surgical skill assessment in laparoscopic colorectal surgery (14). Similarly, Wang and Fey described a deep learning framework for skill assessment in robotic surgery (15). As surgical procedures undergo increased digitalization, coupled with advancements in the automatic and real-time recognition of objects and tasks, the operating room stands as a focal point for progress. The newly developed tools have the capacity to enhance the performance and capabilities of surgical teams.

In the current medical landscape, video plays a crucial role in diagnostic solutions, spanning various applications such as ultrasound, robotic surgery, and endoscopy (16). In contrast to static images, videos offer an effective means to comprehensively depict the entire process and intricacies of diagnosis or surgery. Doctors can grasp the situation and characteristics of lesions or surgical areas in multiple dimensions through time series data. Consequently, the study of video data in the medical field holds considerable significance. In 2022, Zeng et al. proposed Video-Transunet for assisted swallowing therapy in ultrasound video data.8 Based on this, Zeng's team proposed that Video-SwinUnet could also be applied to assist in swallowing therapy diagnosis in ultrasound videos (17). In the field of ultrasound diagnosis, Yeh et al. applied real-time object detection models to assess the recovery of the median nerve in ultrasound videos (18). Yu et al. also applied the U-Net network to the segmentation of surgical instruments in surgical video data for robotic surgery (19). The above research reflects that existing AI models can effectively assist in the automatic localization and segmentation of special targets in ultrasound videos or surgical videos.

Our proposed AI model demonstrated improved performance relative to several other advanced AI models. In evaluation of external cohort, our model equaled the performance of two experienced, senior surgeons with no significant difference (*p* = 0.320, *p* = 0.241; *t*-test). Notably, our model exhibited superior accuracy and precision, albeit with a reduced recall rate. Nevertheless, there are two primary limitations to our model. Firstly, due to the disparities in judgment between the AI model and the surgeons' recognition of PGs, our model might generate more false negatives than surgeons. This observation is evident from the comparison of recall rates between surgeons and our method. Secondly, as videos were recorded subsequent to the initial surgeon's PG identification, there exists the potential for false negatives or overlooked PGs. To mitigate these risks, an experienced surgeon, with over 20 years in the field, validated the PGs in the videos, with additional validation supplied by ICGT. These measures seek to minimize the likelihood of false negatives or missed PGs.

In practical application scenarios, due to the fixed number of PGs, incorrect judgment can easily lead to damage to the correct PG during surgery. Due to our method being more stable in determining the correct PG, it can provide surgeons with a certain degree of judgment advice. Moving forward, our ambition is to apply this method for real-time recognition of PG during surgical procedures. Concurrently, it shall function as an assistive tool for less experienced, junior surgeons in PG recognition, thereby minimizing the incidence of postoperative hypoparathyroidism. We anticipate that these efforts will play an instrumental role in enhancing patient prognosis.

In this retrospective study, we introduced a novel AI-based method to detect the shapes and localization of PGs in thyroid surgery, providing assistance to surgeons in PG detection. Comparative results with surgeons reveal that our method surpasses junior doctors in identifying PGs in intraoperative videos of thyroid surgery and performs at a level equivalent to that of senior surgeons. This provides valuable assistance for surgeons during thyroid surgery.

## Data Availability

The raw data supporting the conclusions of this article will be made available by the authors, without undue reservation.
